# Vitiligo-associated protection against basal cell carcinoma: Clinical observations

**DOI:** 10.1016/j.jdcr.2025.01.041

**Published:** 2025-03-11

**Authors:** Alex Rooker, Marcel W. Bekkenk, Elisabeth H. Jaspars, Rosalie M. Luiten, Walbert J. Bakker

**Affiliations:** a Department of Dermatology and Netherlands Institute for Pigment Disorders, Amsterdam University Medical Center, University of Amsterdam, Amsterdam, The Netherlands; b Cancer Center Amsterdam, Amsterdam Institute for Immunology and Infectious Diseases, Amsterdam, The Netherlands; c Department of Dermatology Amsterdam University Medical Center, VU University, Amsterdam, The Netherlands; d Division of Dermatology, Antoni van Leeuwenhoek – The Netherlands Cancer Institute, Amsterdam, The Netherlands; e Department of Pathology, Amsterdam University Medical Center, University of Amsterdam, Amsterdam, The Netherlands

**Keywords:** autoimmunity, basal cell carcinoma, basal-cell nevus syndrome, bystander lysis, cancer protection, Gorlin syndrome, nevoid basal-cell carcinoma syndrome, skin cancer, vitiligo

## Introduction

Although patients with vitiligo partially lack skin pigmentation, these patients have a lower risk of nonmelanoma skin cancers than healthy controls, particularly basal cell carcinoma (BCC).^1^^,^^2^ In this report, we present 2 cases that underline this remarkable finding. We hypothesize that vitiligo immune reactivity against melanocytes, generally present in BCCs, could lead to BCC tumor destruction via bystander lysis. These patient cases provide clinical evidence of vitiligo melanocyte-specific immune reactions that may inhibit BCC tumor development and/or promote tumor regression, offering insights into the reduced BCC risk observed in patients with vitiligo.

## Case Description

### Basal cell nevus syndrome patient with vitiligo

**Fig 1:**
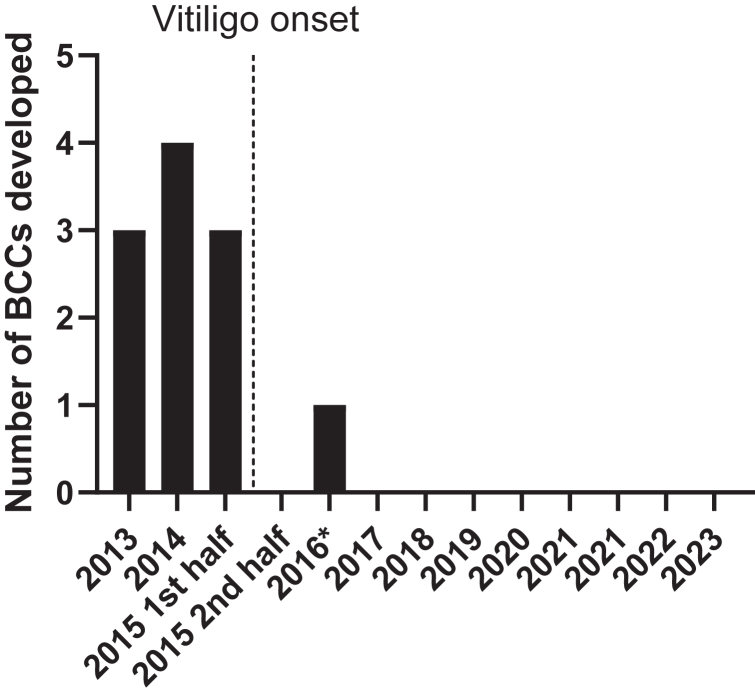
BCC development in the BCNS—patient with vitiligo before and after vitiligo onset. Graph depicts the number of new BCCs that developed per year. ∗BCC located under hairline. *BCC*, Basal cell carcinoma; *BCNS*, basal cell nevus syndrome.

We here describe a 36-year-old woman patient with genetically confirmed diagnosis of basal cell nevus syndrome or Gorlin syndrome since 2013, characterized by frequent and continuous development of multiple BCC lesions. In 2015, the patient developed depigmented skin lesions and was diagnosed with nonsegmental vitiligo. Remarkably, after the onset of vitiligo, she did not develop any new BCC lesions, which persisted during 9 years of follow-up until present (Fig 1). Of note, patients with basal cell nevus syndrome rarely experience a pause of more than a year between new lesions.^3^ The patient did develop new odontogenic keratocysts, indicating ongoing disease activity.

### BCC patient with vitiligo

**Fig 2:**
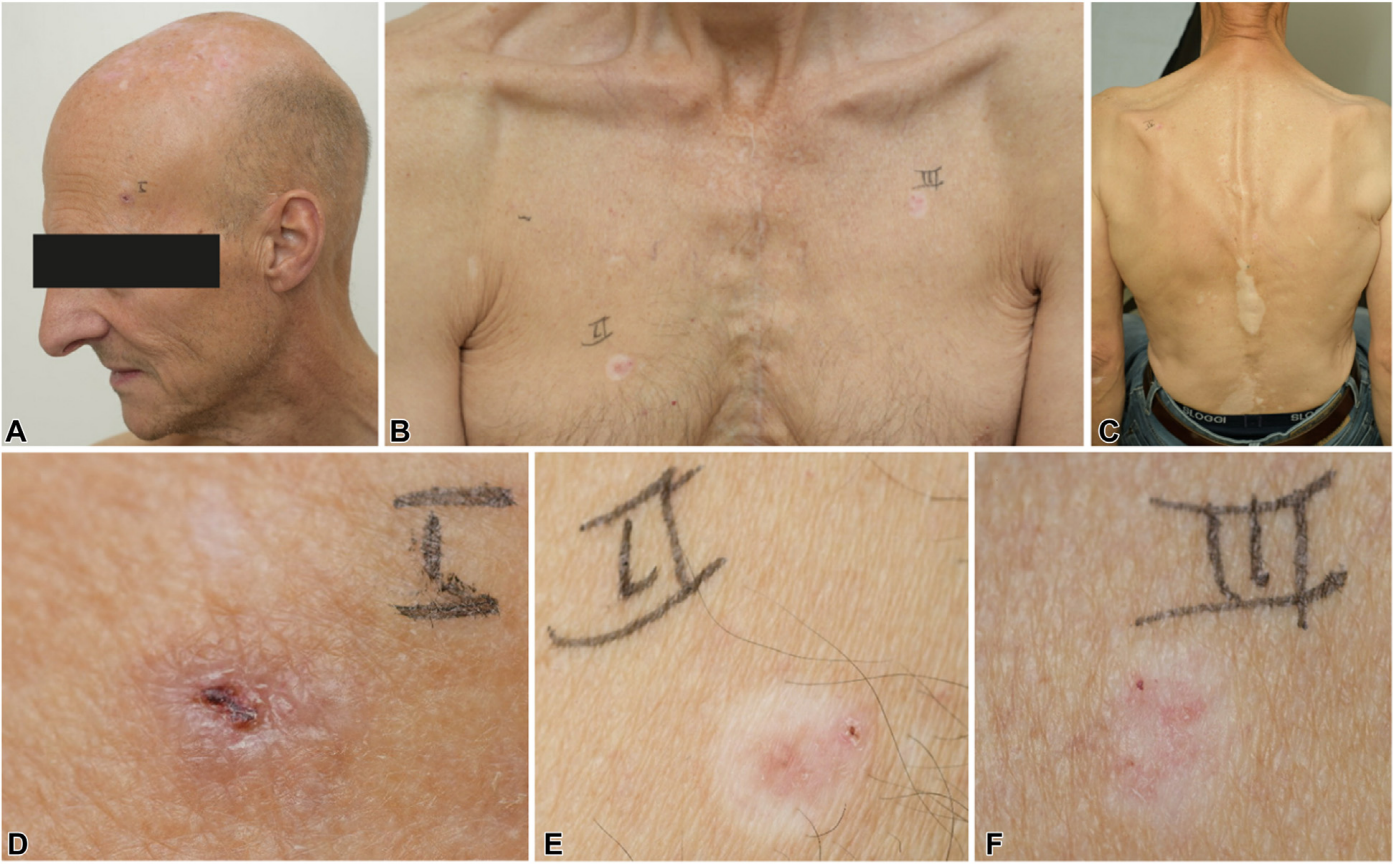
The patient with vitiligo and BCC. A nodular BCC situated on the left temple (**A**) and 2 superficial BCCs located on the right and left side of the chest (**B**). Depigmented lesions on the back indicate vitiligo (**C**). The nodular tumor—I is pigmented (**D**). In contrast, the superficial BCC—II on the right side of the chest (**E**) and superficial BCC—III on the left side of the chest (**F**) show a depigmented halo and signs of regression. *BCC*, Basal cell carcinoma.

A 61-year-old man presented at our dermatology outpatient clinic with several skin lesions (Fig 2, *A, B*). Since the age of 59 years, the patient had developed 3 red lesions on healthy skin and over a 2-year period the patient had experienced a total of 5 BCCs (Table I), a nodular BCC on the left temple (I), 3 superficial BCCs on the right (II) and left (III) sides of the chest and left shoulder blade (IV), and a solid growing BCC on the middle region of the back (V). His medical history includes Hodgkin disease at the age of 28 years, which was treated with total lymph node irradiation (total dose of 36 Gy) resulting in a complete remission. At the age of 48 years, diffuse large B-cell lymphoma (stage IVa) was observed with extensive localization (brain, spleen, bone marrow, thorax, and soft tissue). He was treated with rituximab, cyclophosphamide, doxorubicin, vincristine, and prednisone (R-CHOP) multiagent chemotherapy and local radiotherapy resulting again in a complete remission. Despite fatigue and persisting lymphadenopathy, no relapse was found. His fatigue was due to cardiac problems, possibly induced by the chemotherapy. His dermatologic history reveals nonsegmental vitiligo starting around the age of 30 years (Fig 2, *C*).

**Table I:** Characteristics of excised BCC lesions

Tumor	Location	Type	Melanocytes in lesion∗	Macroscopic halo	Clinical regression
BCC-I	Left temple	Nodular	Yes	No	No
BCC-II	Right side of the chest	Superficial	No	Yes	Yes
BCC-III	Left side of the chest	Superficial	No	Yes	Yes
BCC-IV	Left shoulder	Superficial	nd	nd	nd
BCC-V	Middle region of the back	Superficial	Yes	nd	nd

*BCC*, Basal cell carcinoma; *nd*, not determined.

∗ Melanocyte presence was determined by immunohistochemical staining of Melan-A.

Further examination revealed that the nodular BCC-I on the left temple and solid BCC-V on the middle region of the back presented as a typical pigmented BCC (Fig 2, *D*). In contrast, the 2 superficial BCCs on the right and left side of the chest (II and III) exhibited a depigmented halo (Fig 2, *E, F*). Remarkably, these 2 halo BCCs demonstrated signs of spontaneous regression, in contrast to the pigmented BCC.

**Fig 3:**
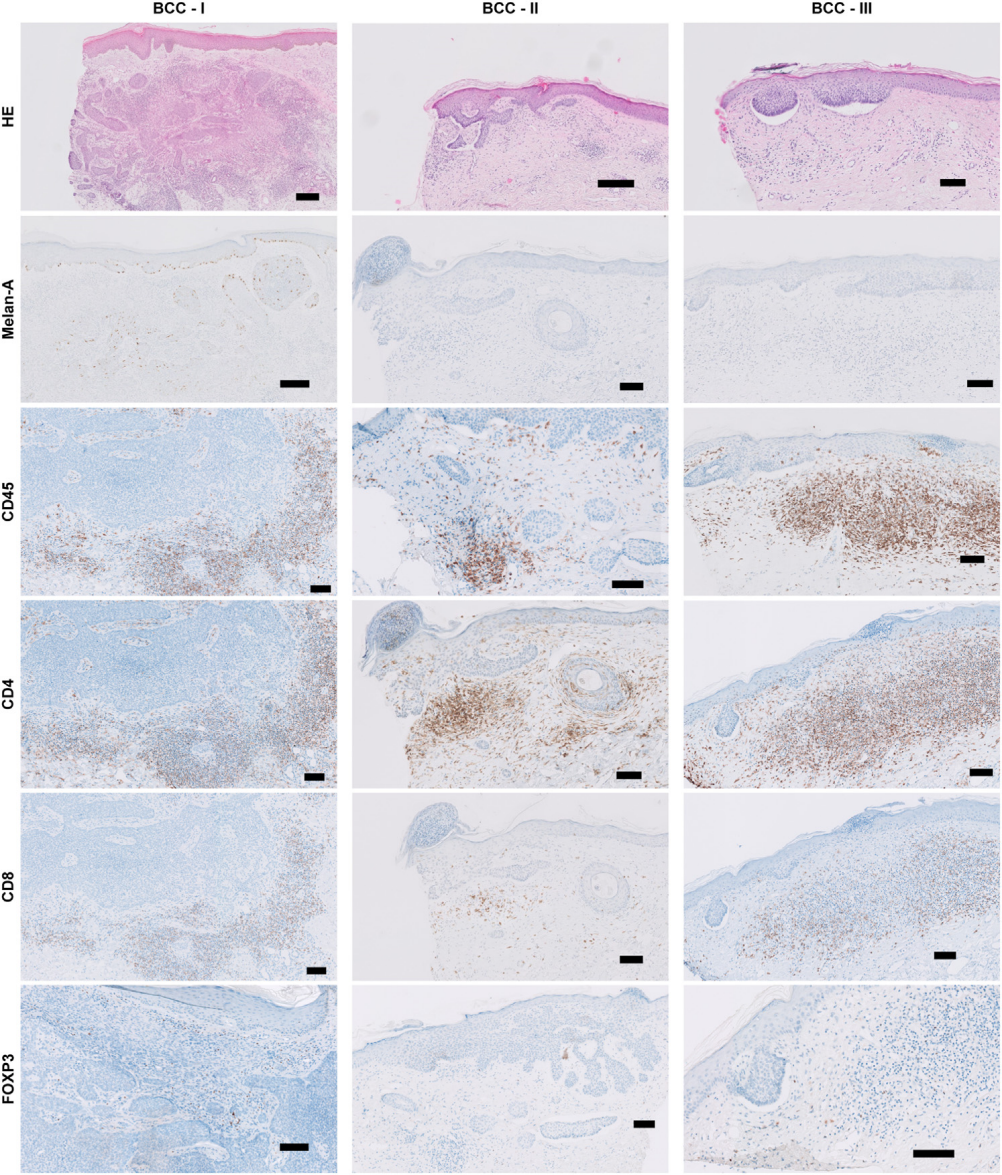
Immune infiltrate in 3 BCC lesions by immunohistochemical staining. Nodular BCC on the left temple—I, superficial BCC on the right side of the chest—II, superficial BCC on the left side of the chest—III. The following stainings were performed: Hematoxylin and eosin (H&E), Melan-A, CD45, CD4, CD8, and FOXP3. *Black bars* are 100 μm, except for BCC—I H&E and BCC—II H&E and Melan-A, *black bars* are 200 μm. *BCC*, Basal cell carcinoma.

Histologic examination of the patient using an hematoxylin-eosin stain of BCC biopsies (I-III) confirmed that the diagnosis of superficial and nodular BCCs. The BCCs (II and III) with clinical signs of spontaneous regression had a macroscopic depigmented halo, and displayed the absence of melanocytes, evidenced by the negative Melan-A staining. In contrast, in the BCC-I biopsies melanocytes were present throughout the epidermis and in the tumor (Fig 3).

To further characterize the immune microenvironment and infiltrate within the BCC lesions, immunohistochemical stainings were performed for CD45 (pan-leukocyte marker), CD3, CD4, and CD8 (T-cell markers), CD20 (B-cell marker), CD57 (natural killer cell marker), and FoxP3 (regulatory T-cell marker). An increase in CD45^+^ immune cells was observed in the tumor-surrounding stroma in all BCCs (Fig 3). B cells and natural killer cells were absent, as indicated by negative CD20 and CD57 stainings (not shown). Both CD4^+^ helper and CD8^+^ cytotoxic T cells were detected, with CD4^+^ T cells being more abundantly present than CD8^+^ T cells. FoxP3^+^ regulatory T cells were found more frequently in the nodular tumor than in superficial tumors, albeit at low numbers compared with the total CD4^+^ T-cell population, suggesting that most CD4^+^ T cells did not have a regulatory phenotype.

## Discussion

Until recently, patients with vitiligo were assumed to have an increased risk of developing skin cancer and were advised to avoid sun exposure. However, epidemiologic meta-analysis data indicates that patients with vitiligo have a decreased risk of developing melanoma and keratinocyte cancers, as compared with healthy controls.^1^ The basal cell nevus syndrome-vitiligo case described here suggests a protective effect of vitiligo against BCC initiation, whereas the BCC-vitiligo case suggests that vitiligo may promote tumor regression.

Immunohistochemical analysis shows substantial immune cell presence in all BCCs. Interestingly, the 2 clinically regressing BCCs lacked melanocytes. Since BCCs in general contain melanocytes, it seems likely that the melanocytes were destroyed by the vitiligo immune reaction.^4^^,^^5^ It has been shown that antigen-negative cells in tumors can be destroyed by T cells via bystander lysis, through secretion of cytotoxic cytokines (eg, interferon gamma) as long as antigen-positive tumor cells are present.^6^ The melanocytes present in BCCs may act as antigen-positive cells, which are recognized by the melanocyte-reactive T cells that mediate vitiligo. This T-cell activation within the BCC tissue may subsequently induce bystander lysis of BCC cells, resulting in tumor regression. The absence of melanocytes in the clinically regressing BCCs is indicative of the hypothesis of bystander lysis. In support of this hypothesis, we previously observed that recognition of melanocytes by vitiligo specific CD8^+^ T cells can induce apoptosis of adjacent (bystander) keratinocytes in a skin explant model.^7^ We found abundant presence of CD4^+^ T cells in the tumor microenvironment (Fig 3), which have been reported to produce cytotoxic interferon gamma and induce senescence of tumor cells.^8^

The remaining BCC that did not show clinical regression, despite immune infiltration and melanocyte presence, might eventually have undergone clinical regression. However, all the examined BCCs were excised and only this snapshot is available.

These 2 cases, provide valuable clinical indications supporting the hypothesis that vitiligo autoimmune reactivity can inhibit BCC initiation and/or growth by the mechanism of bystander lysis.

## Conflicts of interest

None disclosed.
